# Composition and distribution of fatty acids in various lipid fractions in serum and follicular fluid of women undergoing assisted reproductive technology

**DOI:** 10.1371/journal.pone.0286946

**Published:** 2023-06-21

**Authors:** Yujie Liu, Kelly Tilleman, Bruno Vlaeminck, Rachel Gervais, P. Yvan Chouinard, Petra De Sutter, Veerle Fievez

**Affiliations:** 1 Zhejiang Provincial Key Laboratory of Aquatic Resources Conservation and Development, College of Life Science, Huzhou University, Huzhou, PR China; 2 Department of Animal Sciences and Aquatic Ecology, Ghent University, Ghent, Belgium; 3 Department for Reproductive Medicine, Ghent University Hospital, Ghent, Belgium; 4 Department of Biology, Ghent University, Ghent, Belgium; 5 Department of Animal Sciences, Laval University, Québec, Canada; Universita degli Studi di Milano, ITALY

## Abstract

Fatty acids (FA) in follicular fluid (FF) are present in an esterified form [triglycerides, cholesterol esters and phospholipids] or as non-esterified FA, which partly originate from blood. However, a comprehensive comparison of blood vs. FF FA in various lipid classes is missing. The aim of this study was to determine the distribution of the FA composition in each lipid class of serum and FF, and to investigate their mutual correlations. A total of 74 patients undergoing assisted reproductive technology treatment were involved in the study. Both in serum as well as FF, saturated FA and mono-unsaturated FA were predominant in non-esterified FA and triglycerides fractions while poly-unsaturated FA were mainly present in phospholipids and cholesterol esters fractions, although phospholipids also contained high proportions of saturated FA. Irrespective of the lipid class, the FA proportions differed between serum and FF (*P* < 0.05). Despite these differences, most of the FA in triglycerides, phospholipids and cholesterol esters of FF were well correlated with their proportions in serum. Nevertheless, only weak to moderate associations (r < 0.60) were observed for the majority of the FA in the non-esterified FA fraction. Differences in FA product/precursor-ratios were found between serum and FF, such as higher C20:4n-6 to C18:2n-6 and C20:5n-3 to C18:3n-3 in FF. FA metabolism (e.g. desaturation and elongation) takes place in cells of the intrafollicular micro-environment. Moreover, good correlations between esterified FA in serum and FF suggest esterified FA in blood could be representative of esterified FA in FF.

## Introduction

The follicular fluid (FF), the milieu in which the oocyte matures [1], among others constitutes of fatty acids (FA) which have been associated with oocyte maturation, embryo development and subsequent pregnancy [2–7]. Indeed, FF FA play important roles as cellular energy sources, structural components of membranes, and precursors for prostaglandins and steroid synthesis [8]. Several studies have reported direct associations of FF FA in total fats or a specific lipid fraction with reproductive performance [2–7]. Further, FF is the discarded material when oocytes are aspirated from patients undergoing assisted reproductive technology (ART). As such, assessing FF FA has the potential advantage of predicting the oocyte and embryo quality at an early follicular phase. Because of their hydrophobicity, non-esterified FA (NEFA) are bound to albumin in the aqueous FF environment, while esterified FA are included in lipoproteins, with the most hydrophobic components (i.e. triacylglycerols [TG] and cholesterol esters [CHE]) situated in the lipoprotein core. Components having both a hydrophobic and hydrophilic region (cholesterol, phospholipid [PL] and protein) make up the outer surface coat of the lipoprotein [9]. Although lipoproteins in blood include chylomicrons besides very low density lipoproteins (VLDL), low density lipoproteins (LDL), and high density lipoproteins (HDL), only the latter three lipoprotein classes have been reported in FF [10]. Among them, HDL is the smallest and densest class of lipoproteins and is the sole lipoprotein that may intactly pass the follicle basal membrane which acts as a molecular sieve [11]. The albumin-bound NEFA may also pass this molecular sieve. Besides these exudates from plasma, secretions from the follicular granulosa cells also will contribute to the FF composition [12]. Given the various origins of FF FA, assessing which FF lipid classes and individual FA are correlated with blood lipids and FA can provides insights on the impact of external factors (e.g. diet, body mass index, metabolic status) on the FF composition. Nevertheless, to our knowledge, no detailed reports are available on FA in various lipid classes both in FF and blood plasma or serum. Indeed, the focus of former blood versus FF comparisons firstly was on the link between the concentrations in blood and FF of various lipid classes (e.g. Valckx et al. [13]; Pantasri et al. [14]); secondly, on the total FA composition, without distinction in lipid classes (e.g. Kermack et al. [15]) or thirdly, on the FA composition within a specific lipid class (e.g. Jungheim et al. [16]). The studies by Valckx et al. [13] and Pantasri et al. [14] reside within the first group and indicated body mass index-related changes in the concentrations of TG and HDL cholesterol in blood which were also reflected in FF. A dietary intervention study, with marine n-3 FA supplementation contribute to the second group of studies (Kermack et al. [15]). This supplementation resulted in elevated amounts of C20:5n-3 and C22:6n-3 in both red blood cells and FF. Finally, within the third group of studies, Jungheim et al. [16] reported associations between FA in the NEFA fraction of both human blood and FF. However, the number of FA reported in this study was limited, i.e. C18:1n-9, C16:0, C18:2n-6, C18:0 and–although significant–the correlations were weak (R_Pearson_ or R_Spearman_ ranging from 0.236 to 0.309) [16].

More insight in the correlation of blood and FF FA composition within each lipid class allows to assess whether or not the FA composition of specific blood lipid classes reflect the FA composition of this FF lipid class and hence, would allow blood samples to act as an alternative for FF samples. This would be an obvious advantage as blood is easier to obtain than FF and also can be obtained from women not undergoing assisted reproductive technology (ART). As such, the objectives of the present study were: 1) to assess the distribution of the FA composition in each lipid class both in blood serum and FF; 2) to investigate the correlations of FA composition in each lipid class between blood serum and FF.

## Materials and methods

### Participants of the cohort

Women in this study were seeking ART services at Ghent University Hospital (Ghent, Belgium) from 2016 to 2018. The protocol was approved by the local ethics committee of the Ghent University Hospital (2016/0259, Belgian registration number: B670201627735). All participants (n = 74) signed an informed consent prior to ART treatment. The informed consent was obtained from each participant prior to ART treatment. Patients were treated with either an agonist or antagonist protocol, followed by ovum pick-up, in vitro fertilization or intra-cytoplasmic sperm injection, with embryo transfer or vitrification on day 5 of culture, as described by De Croo et al. [17]. The patients’ characteristics and ART outcome were recorded as described in a previous paper [4].

### Sampling of serum and FF

To be eligible for our analysis, women provided a blood serum sample at the same day of oocyte retrieval. Meanwhile, at the time of oocyte retrieval, FF of multiple follicles with diameters between 14 and 24 mm were collected in a single sample. Afterwards, the FF samples were centrifuged at 1500 × g for 10 min, after which the supernatant was stored at -80°C in the biobank of the Ghent University Hospital under the operational management of Bioresource UZ Ghent (ID: BE 71067049) until further analysis.

### Analysis of FA composition in lipids in serum and FF

The FA composition of the non-fractioned extracts was analysed as follows: FA methyl esters (FAME) were prepared from 0.2 mL of FF samples using a direct transesterification procedure described by Vlaeminck et al. [18], with minor modifications. Briefly, toluene (1 mL) containing the internal standard (non-esterified C21:0; Sigma Aldrich, Diegem, Belgium) and methanolic NaOH (2 mL; 0.5 M) were added and the mixture was incubated at 70°C for 60 min. This was followed by 30 min of incubation at 50°C after the addition of methanolic HCl (3 mL), which was prepared by dissolving acetyl chloride in methanol (5/1, vol/vol). The FAME were extracted with hexane.

Prior to FA measurements in different lipid fractions (NEFA, CHE, PL, TG), total FF lipids were extracted following the protocol described in our previous study [19], the extract was separated using the SPE column method, and then methylated according to Valckx et al. [8].

The analysis of FAME was carried out with a gas chromatograph (HP7890A, Agilent Technologies, Diegem, Belgium) equipped with a SP-2560 capillary column (75 m × 0.18 mm × 0.14 μm film thickness, Supelco, Bellefonte, PA, USA) and a flame ionization detector. The temperature program, inlet and detector temperature, carrier gas flow rate, identification of FAME peaks as well as FAME quantification were as described by Liu et al. [4]. Individual FA in non-fractionated extracts and each lipid fractions (NEFA, TG, PL, CHE) were expressed as a percentage of total FA. These proportions were calculated based on both identified and unidentified FA peaks appearing between C12:0 and C22:6n-3. For quantification of FA concentrations, internal standards were used, i.e. C21:0 for non-fractionated extracts, NEFA and PL, and tritridecanoin (C13:0/C13:0/C13:0) for TG and CHE. Also to calculate FA concentrations, both identified and non-identified peaks, in the same region as mentioned before, were used. Concentrations of each lipid fraction (NEFA, TG, PL, CHE) were calculated, as the sum of the individual identified and unidentified FA peaks and expressed as μmol/L. Proportions of each lipid fraction were expressed relative to the sum of the concentrations of the individual lipid fractions.

### Statistical analysis

All statistical analyses were performed with SPSS software version 20.0 (Chicago, IL). The variability among patients of the FA proportions in non-fractionated extracts and in each lipid class of serum and FF were reported as coefficient of variation (CV) for normally distributed data and IQR/Median for non-normally distributed data. Further, FA proportions in non-fractionated extracts and in different lipid fractions were compared between serum and FF by a paired sample t-test or by a Mann-Whitney-Wilcoxon test. Besides, correlations between serum and FF were examined for the proportions of FA in the non-fractionated extracts as well as in each lipid class, using Pearson’s correlation for normally distributed data and Spearman’s correlation for non-normally distributed data. Further, partial correlations between serum and FF, correcting for BMI and age have been checked for some selected, normally distributed FA. However, pearson correlations (without controlling for age or BMI) did not differ from the partial correlations (including BMI and age as covariables). Finally, a principal component analysis (PCA), including all FA data (i.e. FA proportions in the non-fractionated extracts of serum and FF as well as in each of the lipid classes of serum and FF), was performed with SIMCA software, version 17.0.

## Results

### Patient characteristics

Patient characteristics are presented in Table 1. Our data showed that the mean body mass index and age of the patients were 25.1 kg/m^2^ and 32.6 years, respectively. The data of fertility outcome indicated that the mean proportion of expanded blastocysts on day 5 (EB5) to the number of oocytes with two pronuclei (2PN), and the embryo utilization rate (EUR) were 32.8% ± 29.3% and 41.5% ± 27.9%, respectively. The cumulative live birth for the overall patients per cycle was 46.6%.

**Table 1:** Patient characteristics and ART outcome (n = 74).

	Mean ± SD
Body mass index, kg/m^2^	25.1±4.67
Age, year	32.6±4.21
Total doses of urinary gonadotropins, IU	2760±1068
Total doses of recombinant gonadotropins, IU	2076±858
Oocytes retrieved,	15.0±5.65
Oocytes injected + oocytes inseminated,	12.4±5.27
Two-pronuclei oocytes (2PN)^1^,	7.99±4.22
2 pronuclei (2PN) / (oocytes injected + oocytes inseminated), %	65.6±21.6
Embryos with ≥ 6 cells on day 3,	6.14±3.76
Embryos with ≥ 6 cells on day 3 / 2PN, %	77.5±20.9
Embryos with 8 cells on day 3,	3.61±2.94
Embryos with 8 cells on day 3 / 2PN, %	44.4±24.1
Expanded blastocysts on day 5 (EB5), mean ± SD	2.58±2.42
EB5 / 2PN, %	32.8±29.3
Embryo utilization rate (EUR)^2^, %	41.5±27.9
Cumulative live birth rate^3^, %	46.6^3^

^1^ intra-cytoplasmic sperm injection and in vitro fertilization.

^2^ (embryos transferred + number of cryopreserved embryos) / 2PN

^3^ live birth per cycle, expressed per 100 cycle attempts, calculated from the overall participants.

### Concentrations and distribution of lipid fractions in serum and FF

**Fig 1:**
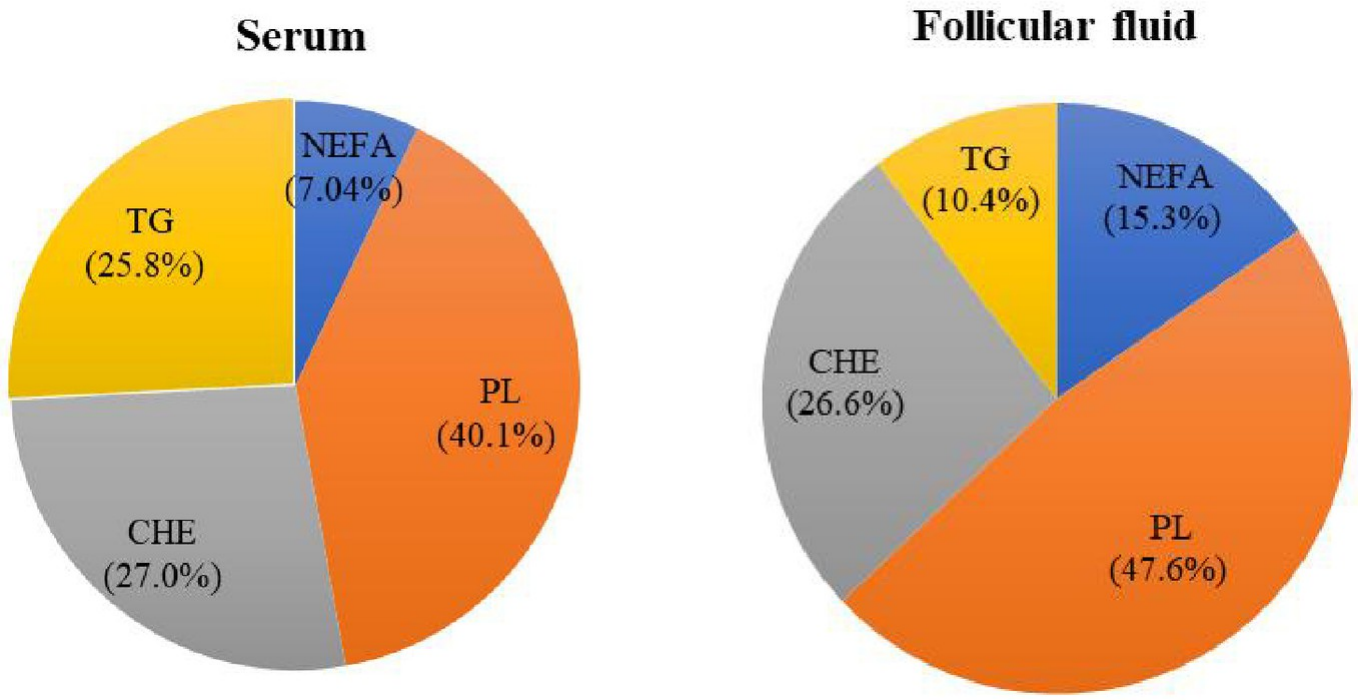
Distribution of lipid fractions in serum and follicular fluid. NEFA: non-esterified fatty acids; TG: triglycerides; PL: phospholipids; CHE: cholesterol esters.

The concentrations of NEFA, PL, CHE and TG in FF were consistently lower than the concentrations measured in serum (S1 Fig). The distributions of different lipid fractions in serum and FF are presented in Fig 1. Phospholipid (PL) were most predominant both in serum and FF, followed by CHE. In serum, 40.1% of the identified FA were present in the PL which reaching up to 47.6% in FF. Slightly more than one quarter of the FA occurred in the CHE fraction, both in serum as well as FF, while another quarter of FA could be attributed to the TG fraction in serum. However, this lipid fraction proportionally decreased around 2.50 times in FF. Contrarily, the NEFA proportion increased 2.17 times in FF as compared with serum.

### FA composition in non-fractionated extracts of serum and FF

The most abundant FA found in total serum and FF lipids were C18:2n-6, C16:0, C18:1n-9, C18:0 and C20:4n-6, and the variability of these FA among individuals were similar in blood and FF, with coefficients of variation ranging from 6.63% to 17.6% (for the non-normally distributed C18:0, the IQR/median ratio is reported) (Table 2). For other FA, the variability among patients was generally higher compared with these predominant FA, with C20:5n-3 showing the highest variation (Table 2). Despite significant differences in the FA composition of total serum and FF lipids (*P* < 0.05 for most FA proportions), variability among patients was similar in serum and FF and the FA proportions in total serum and FF lipids were positively correlated (*P* < 0.05), with most FA showing strong associations (r > 0.60) (Table 2).

**Table 2:** Mean or median (for normally and non-normally distributed data, respectively) percentages of specific fatty acids (% by weight) in serum and follicular fluid (FF), as well as variation among patients in fatty acid proportions within each matrix, followed by a correlation analysis between serum and FF (Rpearson or Rspearman for normally and non-normally distributed data, respectively) (n = 74).

Fatty acids	Serum		FF		*P*-value^3^	R_pearson_ or R_spearman_^4^
	Mean or Median	CV or IQR/Median^2^, %	Mean or Median	CV or IQR/Median^2^, %		
C14:0^1^	0.74	43.8	0.54	41.3	***	0.60
C15:0^1^	0.30	35.4	0.52	40.1	***	0.67
C16:0	22.0	6.68	21.0	6.63	***	0.60
C18:0^1^	6.76	12.0	8.30	9.88	***	0.50
C16:1n-7^1^	1.71	47.2	1.14	38.9	***	0.70
C16:1n-9	0.86	34.6	1.43	57.6	***	0.41
C18:1n-7^1^	1.57	19.2	1.44	14.7	***	0.78
C18:1n-9	21.6	10.6	16.7	9.67	***	0.73
C18:3n-3^1^	0.62	44.7	0.41	32.9	***	0.68
C20:5n-3^1^	0.47	63.1	0.57	58.9	*	0.82
C22:6n-3	1.99	30.0	2.62	27.2	***	0.83
C18:2n-6	27.4	11.8	24.5	12.3	***	0.60
C20:3n-6	1.63	22.8	2.16	22.6	***	0.76
C20:4n-6	6.40	17.6	8.04	17.1	***	0.75
Unknown^1^	3.37	34.4	6.25	45.2	***	0.25
SFA	30.3	4.69	31.5	4.51	***	0.44
MUFA	26.4	9.91	21.4	8.01	***	0.68
n-3 PUFA^1^	3.55	27.9	4.22	30.6	***	0.85
n-6 PUFA^1^	36.2	11.1	36.2	8.69	0.684	0.40
C16:1n-7/ C16:0^1^	0.08	39.2	0.06	34.3	***	0.71
C18:1n-9/C18:0^1^	3.19	24.5	1.97	19.3	***	0.59
C20:4n-6/C18:2n-6	0.24	22.8	0.34	24.3	***	0.81
C20:5n-3/C18:3n-3^1^	0.77	71.3	1.32	83.8	***	0.75
(C20:5n-3+ C22:6n-3)/C18:3n-3^1^	3.77	62.8	7.41	75.9	***	0.76
SFA/MUFA	1.16	10.9	1.48	8.84	***	0.58
n-3/n-6^1^	0.10	28.5	0.12	38.0	***	0.81

Note:

^1^ Data are non-normally distributed.

^2^ For normally distributed data, coefficient of variation (CV) is calculated as the standard deviation/Mean and expressed as percentage. For non-normally distributed data, relative variability is calculated as interquartile range (IQR) / Median and expressed as percentage.

^3^ Paired T-test was conducted for normally distributed data to compare the fatty acid composition between serum and follicular fluid. Otherwise, Mann-Whitney-Wilcoxon test was performed on non-normally distributed data.

***: *P*-value < 0.001.

^4^ Significant correlations for all presented fatty acids.

SFA: saturated fatty acids; MUFA: monounsaturated fatty acids; PUFA: polyunsaturated fatty acids.

Sum of saturated fatty acids (SFA) = Σ (C12:0, C13:0, C14:0, C15:0, C16:0, C17:0, C18:0, C20:0, C22:0, C24:0).

Sum of Monounsaturated fatty acids (MUFA) = Σ (C14:1n-5, C16:1n-9, C16:1n-7, C18:1n-9, C18:1n-7, C20:1n-7, C20:1n-9, C22:1 n-9, C24:1 n-9).

Sum of n-3 Polyunsaturated fatty acids (n-3 PUFA) = Σ (C18:3n-3, C20:3n-3, C20:4n-3, C20:5n-3, C22:5n-3, C22:6n-3).

Sum of n-6 Polyunsaturated fatty acids (n-6 PUFA) = Σ (C18:2n-6, C18:3n-6, C20:2n-6, C20:3n-6, C20:4n-6, C22:4n-6, C22:5n-6).

### The FA composition in NEFA, TG, PL and CHE in serum compared with FF

**Fig 2:**
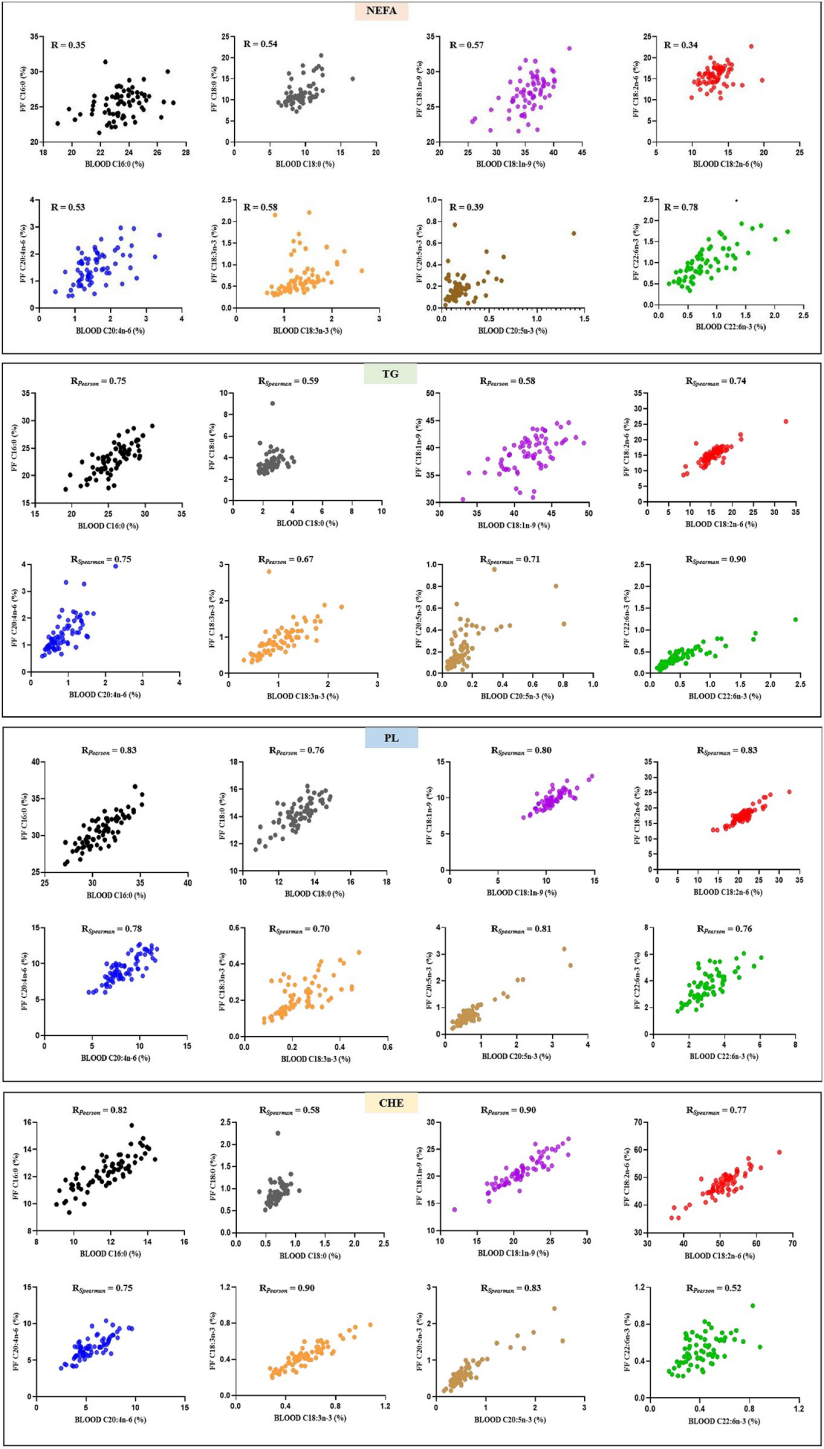
Relationships between blood serum and follicular fluid (FF) of the percentages of a selected number of saturated, mono-unsaturated, n-6 and n-3 poly-unsaturated fatty acids (% by weight) within each lipid class (non-esterified fatty acids, NEFA; triglycerides, TG; phospholipids, PL and cholesterol esters, CHE). R_*pearson*_ or R_spearman_ are mentioned for normally and non-normally distributed data, respectively, and all presented correlations are significant (*P* < 0.05).

The comparison of the FA composition in different lipid fractions in serum and FF is shown in Table 3 and Fig 2. In the separate lipid classes, the predominant FA in serum and FF samples were C16:0, C18:0, C18:1n-9 and C18:2n-6, although their order of importance differed between lipid classes. Saturated FA (SFA) and mono-unsaturated FA (MUFA) were predominant in NEFA, and TG while PUFA were mainly distributed in PL and CHE, although PL also contained high proportions of SFA. More specifically, C16:0 showed high percentages in PL, NEFA and TG fractions while C18:0 was mostly present in PL and NEFA. High percentages of C18:1n-9 were observed in NEFA and TG whereas C18:2n-6, C20:4n-6, C20:5n-3 and C22:6n-3 were predominantly present in PL and CHE. Irrespective of the lipid class, FF showed lower ratios of C16:1n-7 to C16:0 and of C18:1n-9 to C18:0 while higher ratios of C20:4n-6 to C18:2n-6 and C20:5n-3 to C18:3n-3 were observed compared with serum, except for the ratio of C20:4n-6 to C18:2n-6 in the NEFA fraction. Variability within each FA and lipid class among patients were of a similar magnitude in serum and FF. As in the non-fractionated extract, in each lipid fraction, the FA proportions differed between serum and FF (*P* < 0.05). Despite these differences, most of the FA in TG, PL and CHE of FF were well correlated with their proportions in serum. Nevertheless, only weak to moderate associations (r < 0.60) were observed for the majority of the FA in the NEFA fraction.

**Table 3:** Mean or median (for normally and non-normally distributed data, respectively) percentages of specific fatty acids (M, % by weight) in serum and follicular fluid (FF), as well as variation (Var) among patients in fatty acid proportions in 4 lipid fractions (non-esterified fatty acids, NEFA; triglycerides, TG; phospholipids, PL and cholesterol esters, CHE) in serum and follicular fluid (FF), followed by correlation analysis between serum and FF within each lipid class (R refers to R_pearson_ or R_spearman_ for normally and non-normally distributed data, respectively). *P* refers to the *P*-value of the paired T-test or the Mann-Whitney-Wilcoxon test comparing FA proportions of normally or non-normally distributed FA in serum and FF within each lipid class.

**Items**	**NEFA**						**TG**					
	**Serum**		**FF**				**Serum**		**FF**			
**Fatty acids** ^1^	**M**	**Var** ^2^	**M**	**Var** ^2^	***P***	**R** ^3^	**M**	**Var** ^2^	**M**	**Var** ^2^	***P***	**R** ^3^
C14:0	1.82	33.1	1.88	20.4	0.31	0.24	1.68	44.0	1.87	37.5	0.06	0.75
C15:0	0.32	24.1	0.32	39.6	0.82	0.34	0.31	21.3	0.36	23.6	***	0.48
C16:0	23.4	6.34	25.5	7.68	***	0.35	25.7	9.70	23.0	11.8	***	0.75
C18:0	9.26	28.5	10.7	21.4	***	0.54	2.52	20.8	3.32	22.3	***	0.59
C16:1n-7	3.20	35.7	2.32	36.1	***	0.62	2.97	43.4	2.43	41.4	*	0.75
C16:1n-9	0.52	17.6	0.46	25.1	***	0.36	0.89	19.5	0.82	19.6	***	0.71
C18:1n-7	2.08	16.8	2.38	14.9	***	0.43	2.23	20.6	1.90	25.2	***	0.59
C18:1n-9	35.7	8.39	27.0	9.34	***	0.57	42.1	7.55	38.9	8.21	***	0.58
C18:3n-3	1.33	33.4	0.60	64.5	***	0.58	1.04	39.6	0.94	44.1	**	0.67
C20:5n-3	0.16	86.6	0.14	84.1	0.55	0.39	0.11	87.0	0.17	153.8	***	0.71
C22:6n-3	0.72	72.6	0.91	59.4	***	0.78	0.40	93.1	0.34	79.6	0.07	0.90
C18:2n-6	13.3	13.0	15.8	13.9	***	0.34	15.2	20.3	15.3	18.3	0.75	0.74
C20:3n-6	0.27	36.4	0.45	38.1	***	0.65	0.22	42.0	0.36	54.8	***	0.44
C20:4n-6	1.47	47.7	1.43	59.5	0.50	0.53	0.76	74.3	1.31	61.1	***	0.75
Unknown	2.72	39.3	3.20	46.3	***	0.27	0.88	67.2	3.11	70.8	***	NS
SFA	36.3	7.25	41.2	8.34	***	0.31	30.9	9.50	30.8	11.9	0.71	0.59
MUFA	42.9	9.56	33.6	12.1	***	0.50	49.2	6.68	45.4	6.83	***	0.60
n-3 PUFA	2.63	22.5	2.27	29.9	***	0.35	1.84	60.9	1.69	56.2	0.85	0.80
n-6 PUFA	15.6	12.2	19.2	13.8	***	0.41	16.9	20.5	18.0	21.6	*	0.76
C16:1n-7/C16:0	0.14	38.1	0.09	36.3	***	0.53	0.12	38.3	0.11	38.0	*	0.68
C18:1n-9/C18:0	3.95	22.9	2.46	24.9	***	0.51	17.3	21.2	11.6	20.6	***	0.45
C20:4n-6/C18:2n-6	0.12	45.1	0.09	51.8	***	0.44	0.06	41.8	0.10	38.3	***	0.66
C20:5n-3/C18:3n-3	0.13	128.8	0.25	114.7	***	0.37	0.11	91.8	0.20	138.3	***	0.71
(C20:5n-3+C22:6n-3) /C18:3n-3	0.69	93.5	1.89	102.9	***	0.69	0.53	99.2	0.59	91.3	0.33	0.83
SFA/MUFA	0.85	17.5	1.24	19.9	***	0.44	0.63	13.7	0.68	14.6	***	0.49
n-3/n-6	0.17	26.9	0.12	37.2	***	0.54	0.11	52.6	0.1	42	0.18	0.82

**Items**	**PL**						**CHE**					
	**Serum**		**FF**				**Serum**		**FF**			
**Fatty acids** ^1^	**M**	**Var** ^2^	**M**	**Var** ^2^	***P***	**R** ^3^	**M**	**Var** ^2^	**M**	**Var** ^2^	***P***	**R** ^3^
C14:0	0.33	36.3	0.37	33.8	**	0.69	0.78	30.4	0.75	26.1	***	0.89
C15:0	0.28	199.1	0.26	328.6	0.49	0.93	0.21	30.4	0.25	41.2	**	0.32
C16:0	31.0	6.32	30.7	6.89	*	0.83	11.7	11.4	12.4	10.2	***	0.82
C18:0	13.1	7.44	14.2	7.10	***	0.76	0.67	23.4	0.84	21.9	***	0.58
C16:1n-7	0.42	48.6	0.37	34.0	**	0.85	2.45	61.9	1.97	57.3	***	0.89
C16:1n-9	0.16	50.8	0.28	55.9	***	NS	0.45	30.4	0.42	19.7	0.04	0.61
C18:1n-7	1.58	22.9	1.53	17.9	0.32	0.88	1.25	16.8	1.29	18.0	**	0.85
C18:1n-9	10.5	14.1	9.62	15.1	***	0.80	21.0	14.4	20.8	12.7	0.23	0.90
C18:3n-3	0.20	61.4	0.20	62.2	0.29	0.70	0.56	29.6	0.44	28.6	***	0.90
C20:5n-3	0.57	59.2	0.61	62.3	0.20	0.81	0.50	61.9	0.60	71.9	0.09	0.83
C22:6n-3	3.06	32.1	3.65	29.7	***	0.76	0.42	38.5	0.53	29.7	***	0.52
C18:2n-6	21.1	12.0	17.3	13.4	***	0.83	51.4	10.7	47.7	10.5	***	0.77
C20:3n-6	3.07	25.7	3.44	25.9	***	0.96	0.64	27.0	0.76	26.7	***	0.68
C20:4n-6	8.04	33.2	9.16	29.1	**	0.78	5.19	48.7	6.76	30.1	***	0.75
Unknown	3.35	70.5	4.61	61.6	***	0.81	0.96	45.4	1.99	81.8	***	NS
SFA	45.5	3.88	46.6	4.32	***	0.62	13.8	9.60	14.7	10.3	***	0.82
MUFA	13.4	11.2	12.5	10.6	***	0.78	25.1	20.8	24.4	14.9	0.26	0.89
n-3 PUFA	4.37	40.2	5.38	40.0	***	0.70	1.63	52.3	1.88	59.4	**	0.81
n-6 PUFA	33.5	8.14	31.3	8.19	***	0.78	57.9	9.82	56.2	7.60	**	0.74
C16:1n-7/C16:0	0.01	42.6	0.01	35.2	***	0.82	0.21	58.4	0.16	56.2	***	0.89
C18:1n-9/C18:0	0.80	16.6	0.68	19.6	***	0.83	31.6	15.9	24.4	18.4	***	0.44
C20:4n-6/C18:2n-6	0.41	27.2	0.55	25.9	***	0.87	0.11	45.5	0.14	37.3	***	0.78
C20:5n-3/C18:3n-3	2.40	82.0	3.08	88.3	0.143	0.90	0.92	72.7	1.37	74.8	***	0.92
(C20:5n-3+C22:6n-3) /C18:3n-3	16.8	66.8	21.2	67.8	**	0.85	1.61	65.4	2.56	65.5	***	0.90
SFA/MUFA	3.16	47.3	2.24	52.7	***	0.68	0.55	11.3	0.6	11.3	***	0.80
n-3/n-6	9.57	166.2	6.99	113.7	***	0.79	0.03	41.5	0.04	47.6	***	0.81

Note:

^1^ In NEFA fraction, the data of C14:0, C15:0, C18:0, C16:1n-7, C16:1n-9, C18:3n-3, C20:5n-3, C22:6n-3, C20:4n-6, total MUFA, total n-3 PUFA, C16:1n-7/C16:0, C20:4n-6/C18:2n-6, C20:5n-3/C18:3n-3, (C20:5n-3+C22:6n-3) /C18:3n-3, SFA/MUFA and n-3/n-6 are non-normally distributed.

In TG fraction, the data of C14:0, C18:0, C16:1n-7, C20:5n-3, C22:6n-3, C20:3n-6, C20:4n-6, Unknown, total n-3 PUFA, total n-6 PUFA, C16:1n-7/C16:0, C20:5n-3/C18:3n-3, (C20:5n-3+C22:6n-3) /C18:3n-3 and n-3/n-6 are non-normally distributed.

In PL fraction, the data of C14:0, C15:0, C16:1n-7, C16:1n-9, C18:1n-7, C18:1n-9, C18:3n-3, C20:5n-3, C18:2n-6, C20:4n-6, Unknown, total n-3 PUFA, C16:1n-7/C16:0, C18:1n-9/C18:0, C20:5n-3/C18:3n-3, (C20:5n-3+C22:6n-3) /C18:3n-3, SFA/MUFA and n-3/n-6 are non-normally distributed.

In CHE fraction, the data of C15:0, C18:0, C16:1n-7, C16:1n-9, C20:5n-3, C18:2n-6, C20:4n-6, Unknown, total MUFA, total n-3 PUFA, total n-6 PUFA, C16:1n-7/C16:0, C20:4n-6/C18:2n-6, C20:5n-3/C18:3n-3, (C20:5n-3+C22:6n-3) /C18:3n-3 and n-3/n-6 are non-normally distributed.

^2^ For normally distributed data, variation among patients is calculated as the standard deviation/Mean and expressed as percentage. For non-normally distributed data, relative variability is calculated as interquartile range (IQR) / Median and expressed as percentage.

*: *P*-value < 0.05;

**: *P*-value < 0.01;

***: *P*-value < 0.001.

^3^ Except for non-significant (NS) parameters, other fatty acids showed significant correlations.

SFA: saturated fatty acids; MUFA: monounsaturated fatty acids; PUFA: polyunsaturated fatty acids.

Sum of saturated fatty acids (SFA) = Σ (C12:0, C13:0, C14:0, C15:0, C16:0, C17:0, C18:0, C20:0, C22:0, C24:0).

Sum of Monounsaturated fatty acids (MUFA) = Σ (C14:1n-5, C16:1n-9, C16:1n-7, C18:1n-9, C18:1n-7, C20:1n-7, C20:1n-9, C22:1 n-9, C24:1 n-9).

Sum of n-3 Polyunsaturated fatty acids (n-3 PUFA) = Σ (C18:3n-3, C20:3n-3, C20:4n-3, C20:5n-3, C22:5n-3, C22:6n-3).

Sum of n-6 Polyunsaturated fatty acids (n-6 PUFA) = Σ (C18:2n-6, C18:3n-6, C20:2n-6, C20:3n-6, C20:4n-6, C22:4n-6, C22:5n-6).

**Fig 3:**
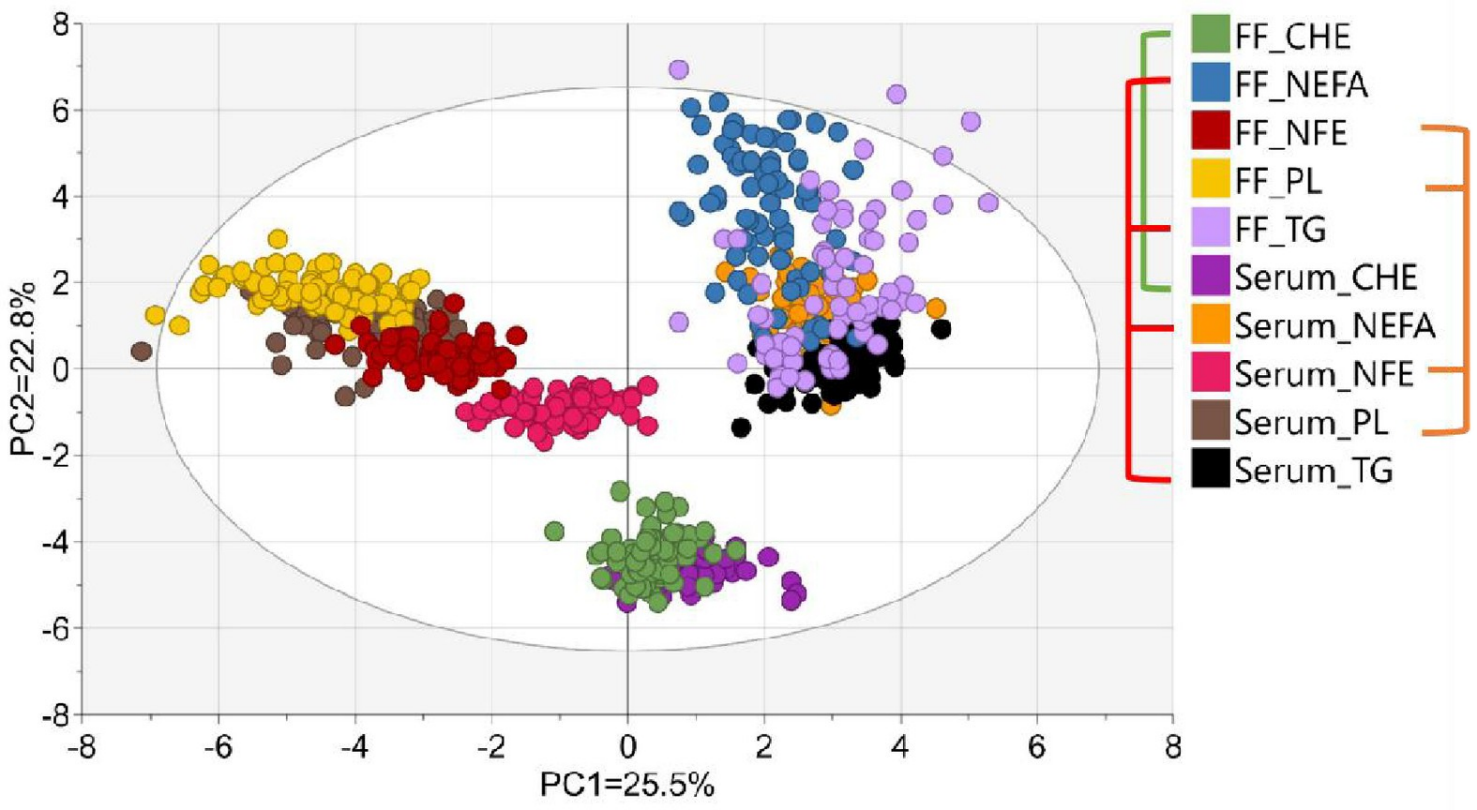
Scatter plot of PCA including the proportions of all identified fatty acids in the non-fractionated extract and 4 lipid fractions of serum and follicular fluid. NFE: non-fractionated extract; NEFA: non-esterified fatty acids; TG: triglycerides; PL: phospholipids; CHE: cholesterol esters.

A PCA was conducted including all identified FA in the non-fractionated extract as well as in the lipid fractions in serum and FF, with the first principal component explaining 25.5% and the second 22.8% of the original variance (Fig 3). This analysis revealed the clustering of serum and FF samples originating from the same lipid class. Moreover, serum and FF NEFA and TG classes clustered at the upper right of the ellipse, while the non-fractioned extracts of serum and FF grouped with the predominant PL class at the upper left of the ellipse. The CHE class of serum and FF merged in a third group which was clearly separated from the other lipid fractions.

## Discussion

Follicular fluid (FF) lipid and FA composition vary with the developmental stage of the ovarian follicle [20], which is related to differences in functions of lipids and FA: i) as constituents of cell membranes (PL) with an important role in signal transduction [21], ii) as major parts of lipid droplets (TG, CHE and NEFA), thereby substantially contributing to energy storage [22, 23], and iii) as major supplier of cholesterol which is an essential precursor in the steroidogenic pathway [24]. From a biological perspective, it would be of most interest to directly link lipid and FA composition of the human oocyte or embryo to their quality. Obviously, destructive analyses, such as lipid extraction on these small matrices show serious limitations, both for ethical as well as technical reasons. Indeed, only unfertilized and discarded human oocytes would be available for such studies [25, 26]; while from a technical point of view, lipid mass of a single oocyte is well below the sensitivity of the analytical method [25]. Consequently, the analysis of compounds (such as lipids and FA) in the follicular micro-environment has been proposed as an alternative tool to associate with oocyte and embryo outcome [2–7].

Generally, serum lipid classes clustered with their counterparts in the FF in a PCA including the proportions of all identified FA from all lipid classes as well as from the non-fractionated extract. Moreover, the considerably lower PUFA proportion in the NEFA and TG classes as compared with the PL and CHE resulted in a grouping of those former lipid classes, away from the PL and CHE. The n-3 PUFA proportion is highest in the PL fraction, while the CHE fraction is particularly enriched in n-6 PUFA, both in serum as well as FF. As such, major differences in FA composition between lipid classes are respected in both body fluids. However, the FA concentrations in total FF lipids were lower than in serum, resulting in FF/serum ratios below 1. Within the group of the esterified FA, the PL fraction showed the highest ratio, followed by CHE and TG, while the NEFA ratio exceeded the ratios of the former 3 lipid classes. Valckx et al. [13], Pantasri et al. [14] and Jungheim et al. [16] also observed lower concentrations of NEFA, TG and cholesterol in FF as compared with blood serum. This could be related to the mechanism of transfer from the blood to FF as well as to the uptake of FA from the FF by the cumulus-oocyte complex. Lipids and FA are building blocks of cellular membranes, are important nutrients to fulfil the oocyte’s energy requirements, fulfil important signalling functions during oocyte maturation and even are critical during the final step of oocyte development which particularly relies on FA oxidation for energy supply [27]. Consequently, there is a high uptake rate of FA from the FF to the cumulus-oocyte complex. Accordingly, the reduced FA concentrations in FF as compared with serum might be partially caused by a high removal from the FF by the cumulus cells. Furthermore, certain lipids and FA are selectively transported from blood into the ovarian follicle [13, 14, 16]. Besides, the blood has a more diverse pool of lipoproteins (including chylomicrons, VLDL, LDL, HDL) than FF, predominantly containing HDL [28, 29]. The highest FF/blood ratio of PL could be explained by the fact that blood HDL are the sole lipoproteins able to cross the follicular barrier [11]. On the other hand, chylomicrons and VLDL mainly contain TG [30]. As these larger blood lipoproteins are not directly transferred from the blood to the FF, it is not surprising that the lowest FF/blood ratio is observed for TG. NEFA are for their part transported in FF by means of albumin, which is smaller in size than HDL and can therefore easily penetrate the follicular barrier [20], which could be related to its relatively high FF/blood ratio. Nevertheless, FA proportions within the NEFA fraction of the FF and serum were only weakly to moderately correlated. This suggests blood NEFA are unlikely to be the sole source of FF NEFA. Indeed, in their comprehensive overview, Hennet and Combelles [31] indicated that metabolites in the FF of the antral follicle originate both from serum as well as metabolic activity of somatic follicular cells. As such, FF NEFA could be expected to partially originate from the metabolic activity in granulosa cells, which potentially may result in a release of NEFA. This additional source might play a role in the poorly correlated NEFA profile in the serum and FF. In contrast to the NEFA fraction, most of the esterified FA in FF showed good associations with serum. Age and BMI may affect the FF FA concentration and composition [4]. However, this does not imply an effect of these factors on the blood FA—FF FA–relationship. Indeed, results of the partial correlations analysis revealed neither age nor BMI changed the relation between blood and FF FA proportions (data not shown). Furthermore, the ratios of C16:1n-7 to C16:0, C18:1n-9 to C18:0, C20:4n-6 to C18:2n-6 and C20:5n-3 as well as (C20:5n-3 + C22:6n-3) to C18:3n-3 differed between serum and FF. This could indicate conversion of the saturated or shorter chain precursor FA to their more unsaturated (and elongated) counterparts. Indeed, several genes related to FA desaturation and elongation, like stearoyl-CoA desaturase, FA desaturase and FA elongase, are expressed in granulosa cells [32, 33]. Stearoyl-CoA desaturase is a rate-limiting enzyme in the cellular synthesis of MUFA from SFA [34], while the enzymes Δ5 desaturase and Δ6 desaturase—encoded by FA desaturase 1 and FA desaturase 2, respectively—are the rate-limiting enzymes in the desaturation of C18:2n-6 to C20:4n-6, and C18:3n-3 to C20:5n-3 and C22:6n-3 [35], and FA elongase is involved in the elongation of long-chain PUFA [36]. Generally, except for C20:4n-6/C18:2n-6 in the NEFA fraction, the product/precursor ratio of the n-3 as well as the n-6 PUFA is elevated in the FF as compared with the serum, which suggests intrafollicular desaturation and elongation of these FA in line with the FA metabolism controlled by the formerly mentioned genes in granulosa cells. As both the ratio of C20:4n-6 to C18:2n-6 and C20:5n-3 (+ C22:6n-3) to C18:3n-3 are higher in FF as compared with serum, interindividual differences in gene expression in the intrafollicular cells could have partially contributed to interindividual differences in FF FA composition. Although endogenous conversion of SFA to MUFA could take place in the granulosa cells through the presence of the enzyme SCD, the ratios of C16:1n-7 to C16:0 and of C18:1n-9 to C18:0 are lower in FF than serum. Potentially, a selective and extensive uptake of MUFA from the FF by cumulus cells could have contributed to their lower proportion in FF as compared with serum. This could be of physiological importance as MUFA have shown to potentially counteract negative effects of saturated NEFA [34]. Indeed, in vitro supplementation of non-esterified palmitic (C16:0) and stearic (C18:0) acids were suggested to inhibit of the expansion of cumulus cells resulting in impaired embryo quality [37].

## Conclusion

This study indicated a distinct FA composition of the different lipid fractions in FF, which also differed from blood. This could be partially related to the selective transport through the blood-follicle membrane of FA in HDL, a lipoprotein of which the lipid fraction particularly consists of PL. Furthermore, differences between serum and FF FA product/precursor-ratios suggest intrafollicular FA metabolism, including elongation and desaturation and resulting in higher C20:4n-6, C20:5n-3 and C22:6n-3 in FF. However, most of the FA proportions in TG, PL and CHE of FF were well correlated with their proportions in serum, while only weak to moderate associations were observed for the majority of the FA in the NEFA fraction. Good correlations between esterified FA in blood and FF suggest the esterified FA composition of blood could reflect esterified FF FA. This may be helpful in future research on the impact of (dietary) FA on oocyte and embryo quality in which blood esterified FA could be monitored instead of FF esterified FA.

## Supporting information

S1 Fig Concentrations of non-esterified fatty acids (NEFA), triglycerides (TG), phospholipids (PL) and cholesterol esters (CHE) in serum and follicular fluid. Note: The concentrations of different lipid fractions between serum and follicular fluid (FF) were compared by a paired T-test or Mann-Whitney test. The data of NEFA, PL, CHE were normally distributed, which allowed a paired T-test comparison, while the data of TG were non-normally distributed, and were compared by a Mann-Whitney test. *P*-value < 0.05 for all comparisons, indicated by *.
(PDF)
