# Supplementary material for: Composition and distribution of fatty acids in various lipid fractions in serum and follicular fluid of women undergoing assisted reproductive technology
Source: PLoS One. 2023 Jun 21;18(6):e0286946. doi: 10.1371/journal.pone.0286946 (PMC10284406; doi:10.1371/journal.pone.0286946)
Supplement: S1 Fig — Note: The concentrations of different lipid fractions between serum and follicular fluid (FF) were compared by a paired T-test or Mann-Whitney test. The data of NEFA, PL, CHE were normally distributed, which allowed a paired T-test comparison, while the data of TG were non-normally distributed, and were compared by a Mann-Whitney test. P-value < 0.05 for all comparisons, indicated by *. (PDF) [file pone.0286946.s001.pdf]

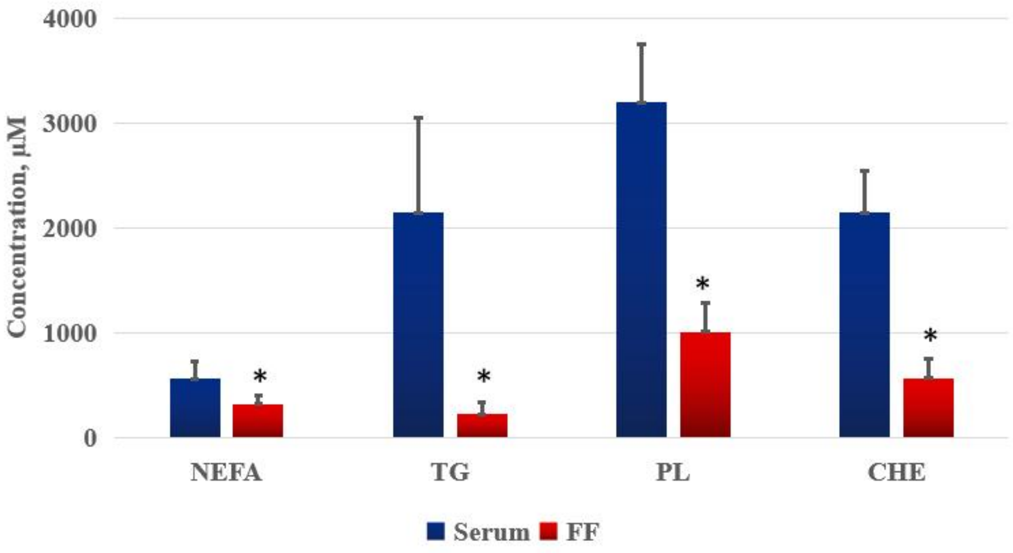

S1 Fig . Concentrations of non-esterified fatty acids (NEFA), triglycerides (TG), phospholipids (PL) and cholesterol esters (CHE) in serum and follicular fluid

Note: The concentrations of different lipid fractions between serum and follicular fluid (FF) were compared by a paired T-test or Mann-Whitney test. The data of NEFA, PL, CHE were normally distributed, which allowed a paired T-test comparison, while the data of TG were non-normally distributed, and were compared by a Mann-Whitney test. *P*-value < 0.05 for all comparisons, indicated by *.
